# BNP and NT-proBNP as Diagnostic Biomarkers for Cardiac Dysfunction in Both Clinical and Forensic Medicine

**DOI:** 10.3390/ijms20081820

**Published:** 2019-04-12

**Authors:** Zhipeng Cao, Yuqing Jia, Baoli Zhu

**Affiliations:** Department of Forensic Pathology, School of Forensic Medicine, China Medical University, Shenyang 110122, China; zpcao@cmu.edu.cn (Z.C.); jia214@hotmail.com (Y.J.)

**Keywords:** BNP, NT-proBNP, heart failure, cardiac dysfunction, forensic medicine, postmortem biochemistry

## Abstract

Currently, brain natriuretic peptide (BNP) and *N*-terminal proBNP (NT-proBNP) are widely used as diagnostic biomarkers for heart failure (HF) and cardiac dysfunction in clinical medicine. They are also used as postmortem biomarkers reflecting cardiac function of the deceased before death in forensic medicine. Several previous studies have reviewed BNP and NT-proBNP in clinical medicine, however, few articles have reviewed their application in forensic medicine. The present article reviews the biological features, the research and application status, and the future research prospects of BNP and NT-proBNP in both clinical medicine and forensic medicine, thereby providing valuable assistance for clinicians and forensic pathologists.

## 1. Introduction

More than 26 million people all over the world are suffering from heart failure (HF) and cardiac dysfunction, which are currently serious global public health problems. The global burden of HF and cardiac dysfunction is increasing rapidly and substantially with the aging of the population [1,2,3,4,5,6]. Due to high morbidity and mortality, the diagnosis of HF and cardiac dysfunction is extremely important in both clinical and forensic medicine [7,8,9,10]. For inpatients, the diagnosis of HF and cardiac dysfunction can be combined with clinically assisted examinations, such as electrocardiography or echocardiography. However, for the deceased examined by forensic pathologists, the diagnosis of HF or the evaluation of cardiac function after death is very difficult due to the lack of clinical medical records of the deceased and unavailability of assisted examinations. Postmortem assessment and diagnosis, especially for HF or cardiac dysfunction of the deceased without typical visible morphological changes, are extremely challenging [10].

Brain natriuretic peptide (BNP) and *N*-terminal proBNP (NT-proBNP) are widely used as significant indicators for the clinical diagnosis of HF and cardiac dysfunction [11,12,13,14,15,16,17]. In recent years, many forensic studies have demonstrated that BNP and NT-proBNP could be used to reflect the cardiac function of the deceased before their death through extensive animal experiments and postmortem specimens, and they could also be used as postmortem biomarkers for the diagnosis of HF or cardiac dysfunction in forensic medicine [9,10,18,19,20]. However, few articles have reviewed application of BNP and NT-proBNP in forensic medicine. For this purpose, this article reviews the biological features, the clinical and forensic research, and the application status of BNP and NT-proBNP, as well as their future research prospects in order to provide valuable assistance for clinicians and forensic pathologists.

## 2. Biological Features of BNP and NT-proBNP

The natriuretic peptide family mainly includes atrial natriuretic peptide (ANP), which is mostly synthesized and secreted by atrial myocytes, BNP, and C-type natriuretic peptide (CNP) [21]. BNP was originally isolated from pig brain tissue in 1988 and was named brain natriuretic peptide, but subsequent studies have shown that its synthesis and secretion are mainly in ventricular myocytes [22].

### 2.1. Structure, Synthesis, and Secretion of BNP and NT-proBNP

BNP is mainly synthesized and secreted by myocytes in the left ventricle (LV) as a response to myocytes stretched by pressure overload or volume expansion of the ventricle [12,23,24,25,26]. The structure of BNP is highly conserved among different species, and the difference between different species is in the length and amino acid composition of the *N*-terminal and *C*-terminal tail chains [27]. Human BNP is a 32 amino acid polypeptide containing a 17 amino acid ring structure with a disulfide bond connecting two cysteine residues [28,29]. The human gene encoding BNP is located on chromosome 1, and the mRNA encoding BNP contains an unstable repeat TATTTAT sequence [28,30,31]. Instead of storage in normal physiological myocardial tissue, the transcription of BNP mRNA and the synthesis and secretion of BNP protein occur in an explosive way and are rapidly released into surrounding tissues after myocardial synthesis [30,32]. Under pathological conditions, the unstable mRNA can rapidly synthesize a 134 amino acid BNP precursor (pre-proBNP) and remove the *N*-terminal 26 amino acid signal peptide to form a 108 amino acid BNP (proBNP), and then, proBNP is split by the proNP convertases, corin or furin, into an inactive 76-amino acid NT-proBNP and an active 32-amino acid BNP [24,33]. Both the biologically active BNP and NT-proBNP could be found in plasma [34,35].

### 2.2. Receptors of Natriuretic Peptides

There are three membrane-bound natriuretic peptide receptors (NPR) for natriuretic peptides, namely NPR-A, NPR-B, and NPR-C. NPR-A is abundant in the vascular endothelium system and some other organs such as kidney and brain [12,34]. NPR-A receptor is the main effector of both ANP and BNP actions, whereas the NPR-B receptor mediates CNP effects. The cyclic guanylate monophosphate (cGMP) levels increase after activation of NPR-A and NPR-B [33,36]. After binding with NPR-A, BNP mediates its biological activities working against the renin–angiotensin–aldosterone system (RAAS) and sympathetic nervous system, improving the glomerular filtration rate and filtration fraction and having diuretic, natriuretic, and vasodilatory effects [37,38].

### 2.3. Degradation of BNP and NT-proBNP

NPR-C is considered by the majority of physiological data to be the receptor mediating internalization and degradation process of clearing natriuretic peptides from the extracellular environment [39]. In addition to NPR-C receptors involved in the degradation of BNP, neutral endopeptidase (NEP), dipeptidyl peptidase-IV (DPPIV), and insulin degrading enzyme (IDE) are also associated with the clearance of BNP under physiological conditions, which leads to an approximate half-life of 20 min for BNP and 90–120 min for NT-proBNP [12,34,39,40]. In 2015, the first of a new class of drugs was approved by the Food and Drug Administration (FDA) of America; it was a sodium supramolecular complex with an equal ratio of the angiotensin receptor blocker valsartan and the neprilysin inhibitor prodrug sacubitril, and it has been proven to be able to successfully cut down mortality in patients suffering from heart failure with reduced ejection fraction (HFrEF) [33,41,42].

## 3. Regulation of BNP Gene Expression

**Figure 1:**
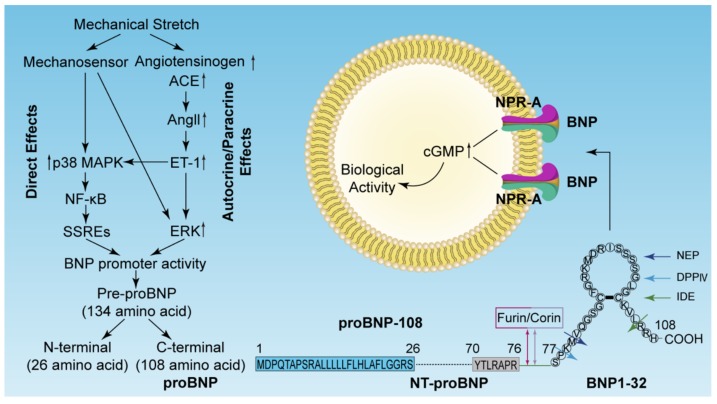
Diagrammatic sketch of mechanical stretch inducing brain natriuretic peptide (BNP) signal transduction events, structural processing, receptor binding, cleavage processing, and degradation enzymes (modified from [36,43] with permission).

The synthesis and secretion of BNP can be induced by mechanical stress, systemic ischemia and hypoxia, neurohumoral factors, and more. However, the exact mechanism of complete regulation remains unclear. It is now generally accepted that mechanical stretch is the main cause of BNP rise in the myocardium. After mechanical stress acts on cardiomyocytes, BNP may be induced by an endothelin (ET)-independent or an ET-dependent pathway [36,43] (Figure 1).

### 3.1. ET-Independent Pathway (Direct Effects)

Mechanical stress signals act on mechanosensors. Then, signaling from the extracellular matrix through integrin activates the mitogen-activated protein kinase (MAPK) signaling pathway, thereby activating the BNP promoter [43]. BNP production induced by mechanical stress is mainly dependent on p38 MAPK, which is a subtype of MAPK. The activated p38 MAPK continues to activate its downstream nuclear factor kappa B (NF-κB), which binds NF-κB to shear stress-responsive elements (SSREs) in the BNP gene promoter, thereby enabling BNP gene promoter activation [43]. p38 MAPK has four subtypes: α, β, γ, and δ. Among them, p38α induces BNP gene transcription through activator protein-1 (AP-1), while p38β regulates BNP gene expression through ET-1-induced transcription factor GATA-4 [33,44]. GATA-4 and many other transcriptional regulators, such as nuclear factor of activated T-cells, myocardin, serum response factor, and more, have been shown to be transcriptional effectors that regulate the transcription of BNP [33].

### 3.2. ET-Dependent Pathway (Autocrine/Paracrine Effects)

While stress receptors activate intracellular kinases, mechanical stress stimulates the formation of angiotensin II (Ang II) and ET-1 complexes, which activate BNP gene activation via p38 MAPK and extracellular signal regulated kinase (ERK) signaling pathways [45,46]. Ang II is an octapeptide substance produced by the hydrolysis of angiotensin I (Ang I) under the actions of angiotensin enzyme and is the main response factor of the renin-angiotensin system [47]. Animal studies have demonstrated that the BNP mRNA level in the left ventricle of rats increased to 4.5 times that of the control group after Ang II was injected into rats for 6 h and increased to 1.8 times after two weeks. While, the Ang II type 1 receptor (AT1R) antagonist was administered, the BNP mRNA level in the left ventricle of rats was significantly reduced, which may be related to the decrease of aldosterone. This indicated that Ang II induced BNP production by binding to AT1R [47]. Ang II has also been proven to be able to promote the synthesis of BNP during myocardial fibrosis by inducing ET-1 gene expression [48]. ET is currently the most potent long-acting vasoconstrictor. It is produced by both endothelial cells and cardiomyocytes, and has three isomeric peptides, of which ET-1 conducts the very potent vasoconstriction and smooth muscle contraction by binding to ET-A receptor [49]. ET-1 is also a major cause of cardiovascular disease and has been reported to activate the NF-κB transcription factor, which is mediated by the phosphorylation of p38 MAPK, and also to activate the GATA-4 transcription factor, which regulates the expression of BNP [46].

### 3.3. Other Factors

Some other factors have also been reported to regulate BNP expression but may not be the dominant ones. Natriuretic peptides are frequently increased in primary aldosteronism patients. Aldosterone has been widely proven to be able to active NF-κB, and Ang II is reported to stimulate the synthesis of aldosterone, which can also be suppressed by BNP [50,51,52]. Ang II and aldosterone often collaborate in pathological conditions to induce cardiac fibrosis, hypertrophy of cardiomyocytes, and cardiac remodeling [53]. Thyroid hormone and its receptor levels are decreased in patients with HF and myocardial infarction animal models, suggesting that BNP mediates the pathophysiological mechanism of thyroxine involved in HF and myocardial infarction. Thyroid hormone may trigger hypertrophy in cardiac myocytes, and BNP gene, as a target of thyroid hormone action, increases under the action of thyroid hormone including BNP promoter activity, BNP mRNA, and BNP protein expression levels [54,55]. In cardiac allograft rejection, activated T lymphocytes produce inflammatory factors such as tumor necrosis factor, IL-1, and IL-6, which also selectively upregulate BNP secretion [56].

Various stimuli that cause cardiac hypertrophy, ischemia, and hypoxic damage, such as growth factors, adrenergic receptor agonists (catecholamines), thyroid hormone, Ca^2+^, and more, may act on BNP promoter elements through a variety of signaling pathways and affect the activity of its promoter. The activation and transmission of these signaling pathways are different but could cooperate with each other [56].

## 4. BNP and NT-proBNP as Clinical Biomarkers for the Diagnosis of HF

HF is a multifactorial systemic disease affecting approximately 1 to 2% of the adult population. Cases of HF can currently be divided into HFrEF and “heart failure with normal or preserved ejection fraction” (HFnEF or HFpEF), depending on the ejection fraction (EF) [57,58]. According to the guidelines of the American College of Cardiology Foundation/American Heart Association (ACCF/AHA) and the European Society of Cardiology (ESC), BNP and NT-proBNP are considered to be the most valuable and reliable biomarkers for diagnosing HF and cardiac dysfunction. They are also responsible for the determination of the severity, guiding the relevant treatment strategies, and assessing the prognosis of heart disease [59,60,61,62,63,64].

### 4.1. Clinical Cutoffs of BNP and NT-proBNP

The ESC guidelines for the diagnosis and treatment of acute and chronic HF in 2016 recommends that all patients with suspected acute HF should have their plasma natriuretic peptide levels (BNP and NT-proBNP) tested to help identify acute HF. The upper limit of normal in the non-acute setting for BNP is 35 pg/mL and for NT-proBNP is 125 pg/mL, while in acute setting, the cutoff value for BNP is 100 pg/mL and for NT-proBNP is 300 pg/mL [59]. BNP levels can help clinicians distinguish the cause of dyspnea due to HF or other causes. If BNP < 100 pg/mL, HF is considered unlikely and alternative causes of dyspnea are pursued. If BNP is between 100 and 500 pg/mL, clinical judgment should be used to diagnose HF. If BNP is >500 pg/mL, HF or cardiac dysfunction is considered possible and rapid therapy for HF is suggested [65]. Based on International Collaborative of NT-proBNP (ICON) study, age-dependent cutoffs of NT-proBNP may be more useful for the diagnosis of HF. Acute HF could be excluded with a general age-independent cutoff of 300 pg/mL. However, HF should be diagnosed for patients who are less than 50 years old with NT-proBNP levels > 450 pg/mL, patients who are between 50 and 75 years old with NT-proBNP levels > 900 pg/mL, and patients who are more than 75 years old with NT-proBNP levels > 1800 pg/mL [66].

### 4.2. Diagnostic Role in a Failing Heart

HF and cardiac dysfunction—caused by various causes, such as ischemic heart disease, different types of arrhythmia, and cardiomyopathy—can lead to an increase in BNP and NT-proBNP [29,65,67,68,69,70,71,72,73].

Acute ischemic heart disease is associated with an elevation of BNP levels, which might reflect the severity of LV dysfunction, and studies have suggested using natriuretic peptide levels as a guide to institute more aggressive treatments for ischemic heart diseases aimed at reducing ventricular wall stress [29]. In patients with stable coronary heart disease, both BNP and NT-proBNP are strong predictors of adverse cardiovascular events [67]. BNP and NT-proBNP were evaluated—along with myocardial injury markers cardiac troponin T (cTnT), myoglobin, and creatine kinase MB (CK-MB)—in acute myocardial infarction patients. NT-proBNP, which remained elevated on average for 12 weeks, might be a better diagnostic biomarker than BNP [32,70]. BNP and NT-proBNP are highly sensitive and specific indicators of the size of a myocardial infarction, and they are also valuable markers for predicting the prognosis and severity of ischemic heart disease in patients with acute coronary syndrome [68,69].

Apart from ischemic heart diseases, BNP and NT-proBNP were also reported to be related to arrhythmias and cardiomyopathies. Both BNP and NT-proBNP were found to be increased in atrial fibrillation patients [65]. BNP mRNA and its protein are demonstrated to increase as early as 10 min after transient lethal ventricular arrhythmias in animal experiment [74]. BNP and NT-proBNP correlated directly with left ventricular end-diastolic dimension (LVEDD) and left ventricular volumes and were inversely correlated with left ventricular ejection fraction (LVEF) in patients with dilated cardiomyopathy and hypertrophic cardiomyopathy [71,72,73]. The levels of BNP are significantly high in Takotsubo cardiomyopathy, and early BNP/cTnT and BNP/CK-MB ratios help differentiate Takotsubo cardiomyopathy from acute myocardial infarction (AMI) with greater accuracy than BNP alone [75]. This indicates that the assays of BNP in combination with other biomarkers could be used for the differential diagnosis of certain heart diseases.

### 4.3. Assessing the Severity and Prognosis of HF

BNP and NT-proBNP do not only have a great significance in the diagnosis of HF, but they also have an assistance value for assessing the severity and prognosis of HF. BNP and NT-proBNP were the strongest independent predictors for HFpEF, as determined by Doppler-echocardiography [76]. A designed trial based on the New York Heart Association (NYHA) classification system, in which patients considered to have NYHA classes I–IV were observed to have gradually increasing plasma BNP concentrations, suggesting that plasma BNP concentration increases with the severity of HF [77]. Plasma BNP and NT-proBNP levels have prognostic values in patients with cardiovascular diseases, and the reduction of BNP and NT-proBNP level predicts an improvement in clinical symptoms. There is a positive correlation between the risk of death and evaluated BNP or NT-proBNP [64]. A study of 521 AMI patients found that BNP and NT-proBNP predicted sudden cardiac death and were the strongest predictors, even after adjusting for clinical variables, including EF [78]. Plasma BNP and NT-proBNP are also used clinically to guide the management of patients with HF and cardiac dysfunction, and they are also used as prognostic indicators which can help clinicians adjust their therapy strategy and determine therapy effectiveness to improve a patient’s survival [40,79].

### 4.4. Therapeutic Role in Cardiac Dysfunction

Recombinant human brain natriuretic peptide (rhBNP) is a synthetic endogenous hormone with the same amino acid sequence as BNP. It can directly dilate blood vessels and effectively reduce cardiac preload and afterload. Nesiritide, approved by the FDA for the therapy of acute decompensated HF in 2001, is a successful rhBNP that has several biological functions that are similar to endogenous BNP, including facilitating natriuresis, diuresis, inhibiting RAAS, increasing output of the heart, decreasing wedge pressure in pulmonary capillaries, and improving cardiac diastolic and systolic function [80,81,82,83]. As of today, rhBNP has been widely used for the therapy of HF from various causes.

## 5. BNP and NT-proBNP as Postmortem Biomarkers to Evaluate Cardiac Function in Forensic Medicine

### 5.1. Forensic Significance of Functional Biomarkers

Different from clinicians, forensic pathologists only focus on the diagnostic value of BNP and NT-proBNP. The diagnosis of HF or evaluation of cardiac dysfunction at autopsy is based predominantly on morphological and pathological findings. This includes the venous congestion of multiple organs, such as the lungs and liver, or a systemic low output state with ischemic arterioles and capillaries [84]. Acute cardiac dysfunction caused by early acute ischemic heart disease and fatal arrhythmia has become a difficult problem in the field of forensic science and pathology due to its high incidence and the lack of typical pathological changes [7,85,86]. The visible morphological changes of the myocardial structure caused by acute heart diseases, such as acute myocardial ischemic injury, are quite limited [87]. Objective evidence for the diagnosis of HF or cardiac dysfunction is extremely necessary in forensic medicine. Compared with morphological indicators, functional indicators or biomarkers, such as BNP and NT-proBNP, could reflect the cardiac function and pathophysiological processes during death and may better clarify the mechanism of death in forensic medicine [88]. The functional biomarkers BNP and NT-proBNP played quite an important role in postmortem biochemistry, and could help solve forensic problems in many routine natural deaths [88,89,90,91].

### 5.2. Pericardial Fluid in Postmortem Biochemistry

Being essentially the ultrafiltration of plasma, pericardial fluid is the pale yellow, clear, and transparent liquid present in the pericardial cavity which acts to lubricate and prevent adhesions. The normal pericardial fluid volume in the physiological condition is 20–30 mL [92,93,94]. Compared with the fact that blood and other bodily fluids are susceptible to postmortem changes, such as autolysis and spoilage, pericardial fluid is present in a closed serosa cavity and is not susceptible to contamination and postmortem changes [92,93]. It is easy to obtain during forensic autopsy and acts not only as a clinically important sample but also has wide application prospects in forensic identification. It is currently used as a substitute for serum in postmortem biochemical assays [95,96]. Forensic studies have also reported that ions and protein components in pericardial fluid could be used for forensic identification of sudden cardiac death, mechanical asphyxia, hypothermia, hyperthermia, and death inference [8,10,88,93,97,98,99,100]. Several studies have also reported the postmortem biochemical investigations of BNP and NT-proBNP in pericardial fluid, which were associated with different causes of death [7,8,9,10].

### 5.3. Postmortem BNP and NT-proBNP

As acute or subacute HF may occur in many acute diseases or traumatic deaths, objective evaluation of end-stage cardiac function status has great significance for forensic diagnosis [101]. Unlike other cardiac biomarkers, such as cTnT and cTnI existing in physiological cardiomyocytes, BNP is not stored in normal myocardial tissue under physiological conditions. However, the transcription of BNP mRNA and the synthesis of its protein can occur and accelerate sensitively and rapidly in a very short time under pathological conditions [30,32]. This means that BNP and NT-proBNP do not fluctuate greatly after death and might be more objective biomarkers of cardiac function [102,103]. In the past decade, a few research teams have conducted postmortem BNP and NT-proBNP studies. To investigate BNP and NT-proBNP concentrations in bodily fluids and myocardial tissue, and the expression of BNP mRNA in myocardium may objectively reflect the end-stage cardiac function status of the deceased before death, which are mainly described as below [88].

Studies in postmortem individuals have demonstrated that BNP and NT-proBNP concentrations were significantly elevated in the blood and pericardial fluid of the deceased who died from acute ischemic heart disease (with or without myocardial necrosis), chronic congestive heart disease, arrhythmogenic right ventricular cardiomyopathy, and more. BNP mRNA was also elevated in the myocardium of individuals with these diseases [7,8,90]. The concentration of BNP in pericardial fluid was closely related to the cause of death, and compared with non-cardiac death, the BNP levels were significantly increased in sudden cardiac death cases, such as acute ischemic heart disease and recurrent myocardial infarction. This further confirms that BNP is important for evaluating the cardiac function of the deceased with ischemic heart disease [9]. High levels of BNP and BNP/ANP ratios in pericardial fluid after death are hallmarks of the duration of cardiac dysfunction before death, which may be due to subacute and chronic ventricular dilatation [9]. Patients with arrhythmogenic right ventricular cardiomyopathy have elevated BNP levels in the pericardial fluid but, interestingly, BNP mRNA levels in the right ventricular myocardium are higher than those in the left ventricular myocardium [7,8]. BNP protein and mRNA were also demonstrated to be elevated in acute cardiac dysfunction caused by acute ventricular arrhythmias, indicating that BNP may be of great forensic significance in the diagnosis of acute cardiac dysfunction without any morphological changes [74]. In some forensic cases of death which are difficult to distinguish from sudden cardiac death, such as hemopericardium and pulmonary thromboembolism, neither BNP levels in the pericardial fluid nor BNP mRNA levels in the myocardial tissue increased, indicating that BNP and BNP mRNA can also be used for distinguishing different diagnoses [8].

Furthermore, in forensic medicine, NT-proBNP is expected to be a more reliable postmortem biomarker compared with BNP due to its greater stability and longer half-life of 90–120 min as mentioned above, and it is not susceptible to temperature, storage time, and storage conditions [104,105,106,107,108]. Several studies have focused on the postmortem investigation of NT-proBNP in different bodily fluids. Postmortem concentration of NT-proBNP in serum from femoral blood within 24 h after death has no difference with the antemortem serum NT-proBNP concentration, and it was stable within 48 h. Cardiopulmonary resuscitation before death has been found to have no effect on NT-proBNP results [19]. Serial assays of NT-proBNP in blood and pericardial fluid, which were gathered from corpses with a postmortem interval of up to 24 h, showed that NT-proBNP was stable over 24 days and, particularly, the concentration of NT-proBNP in pericardial fluid decreased by no more than 16% after storage at −20 °C for 24 days [18]. NT-proBNP concentrations in different samples, such as serum and pericardial fluid, reveal good correlations, and NT-proBNP was demonstrated to be much higher in pericardial fluid than other fluids, such as serum, which indicates that the investigation of NT-proBNP in pericardial fluid might be a much better choice in postmortem biochemical assay [18,19].

### 5.4. Limitation of BNP and NT-proBNP in Forensic Medicine

While BNP and NT-proBNP in pericardial fluid are not susceptible to being polluted, serious hemolysis and other postmortem changes caused by various factors, such as the preservation conditions of the corpse, may affect the postmortem biological assays of BNP and NT-proBNP. This should be taken into consideration in postmortem biochemical assays, and the affection of hemolysis can be reduced by the physical filtering of body fluids [109,110]. In addition, valuable postmortem cutoffs are still needed for the further study of both BNP and NT-proBNP in blood or pericardial fluid [89]. Currently, because of equipment and personnel, not every forensic laboratory around the world conducts postmortem biochemical assays, which is one of the reasons for the lack of postmortem cutoffs for BNP and NT-proBNP based on large amounts of data. It is worth mentioning that all diuretics, including blockers of renin angiotensin aldosterone and aldosterone receptor, could decrease BNP levels due to the amelioration of plasma volume and sodium, which should also be considered in forensic utilization of BNP or NT-proBNP.

## 6. Research and Application Prospects in Clinical and Forensic Medicine

BNP and NT-proBNP are currently used in the evaluation of cardiac function status in clinical and forensic practice. In recent years, many studies have confirmed that some non-coding RNAs are highly expressed in patients with cardiac dysfunction and participate in the regulation of BNP expression [111,112,113,114,115]. Therefore, exploring the expression patterns of BNP-related specific non-coding RNAs, such as microRNAs, in clinical and forensic samples and exploring how they regulate the expression of BNP and the expression of non-coding RNA in forensic degradation or corrupted samples may be the future research direction in this field.

In addition, exosomes, the small vesicles in different bodily fluids such as serum and urine, have been proven to be contained in different molecules such as proteins, DNA, and RNA (coding RNA and non-coding RNA). Exosomes have been expected to be a new hot issue in the field of markers for cardiovascular diseases due to their specific diagnostic value and unknown underlying mechanisms [116,117,118]. Exosome RNA and proteins are demonstrated to be related to cardiac dysfunction and mediate cardioprotective abilities [119,120,121,122]. Previous studies have found that exosomes containing AT1Rs were isolated from mice undergoing cardiac pressure overload. However, few studies have reported whether exosomes in bodily fluids were correlated to BNP. Thus, exosomes as a biomarker for diagnosing cardiac dysfunction in clinical and forensic medicine may also be a future research prospect [122]. As written above, pericardial fluid is an ideal biological sample for forensic pathology. Clinical research has proven that exosomes in human pericardial fluid are diagnostic and therapeutic molecules for heart disease. Whether exosomes in pericardial fluid can be used to diagnose heart disease in forensic medicine still needs to be further studied [123,124,125,126].

Postmortem biochemical assays and molecular biological methods, such as the analysis of mRNA—which should both be taken as the routine laboratory assays in forensic medicine—may be potentially useful for investigating the pathophysiology, process, and the cause of death. They may also offer powerful support by providing visible evidence for pathognomonic assessment, including cardiac function [88]. Therefore, with its advantages in assessing pathophysiological functional changes involved in the dying process, combined assays of postmortem chemistry and molecular biology of BNP and NT-proBNP may better support and reinforce morphological evidence in forensic medicine [90,127,128,129].

## 7. Conclusions

More than 30 years of research has outlined the significant contribution of BNP in cardiovascular disease, particularly in HF and cardiac dysfunction. Based on their important diagnostic, therapeutic, and prognostic roles, BNP and NT-proBNP have been used as important biomarkers in clinical and forensic medicine. With the rapid development of molecular biological technology, the accurate instigation of BNP and NT-proBNP will be better used for the assessment of clinical and forensic cardiac function status in the future.

## Abbreviations

ACCF	American College of Cardiology Foundation
AHA	American Heart Association
Ang	Angiotensin
ANP	Atrial natriuretic peptide
AMI	Acute myocardial infarction
AP	Activator protein
AT1R	Angiotensin II type 1 receptor
BNP	Brain natriuretic peptide
cGMP	Cyclic guanosine monophosphate
CK-MB	Creatine kinase MB
CNP	C-type natriuretic peptide
cTn	Cardiac troponin
DHF	Diastolic heart failure
DNA	Deoxyribonucleic acid
DPPIV	Dipeptidyl peptidase-IV
EF	Ejection fraction
ERK	Extracellular signal regulated kinase
ESC	European Society of Cardiology
ET	Endothelin
FDA	Food and Drug Administration
HF	Heart failure
HFnEF	Heart failure with normal ejection fraction
HFpEF	Heart failure with preserved ejection fraction
HFrEF	Heart failure with reduced ejection fraction
ICON	International Collaborative of NT-proBNP
IDE	Insulin degrading enzyme
LV	Left ventricle
LVEDD	Left ventricular end-diastolic dimension
LVEF	Left ventricular ejection fraction
NEP	Neutral endopeptidase
NF-κB	Nuclear factor kappa B
NT-proBNP	N-terminal pro-brain natriuretic peptide
NPR	Natriuretic peptide receptor
NYHA	New York Heart Association
MAPK	Mitogen-activated protein kinase
RAAS	Renin–angiotensin–aldosterone system
rhBNP	Recombinant human brain natriuretic peptide
RNA	Ribonucleic acid
SHF	Systolic heart failure
SSREs	Shear stress-responsive elements
